# Understanding cardiopulmonary interactions through esophageal pressure monitoring

**DOI:** 10.3389/fphys.2023.1221829

**Published:** 2023-07-19

**Authors:** Elena Spinelli, Gaetano Scaramuzzo, Douglas Slobod, Tommaso Mauri

**Affiliations:** ^1^ Department of Anesthesia, Critical Care and Emergency, IRCCS (Institute for Treatment and Research) Ca’ Granda Maggiore Policlinico Hospital Foundation, Milan, Italy; ^2^ Department of Translational Medicine, University of Ferrara, Ferrara, Italy; ^3^ Department of Critical Care Medicine, McGill University, Montreal, QC, Canada; ^4^ Department of Pathophysiology and Transplantation, University of Milan, Milan, Italy

**Keywords:** transpulmonary pressure, hemodynamics, mechanical ventilation, pleural pressure, heart-lung interaction

## Abstract

Esophageal pressure is the closest estimate of pleural pressure. Changes in esophageal pressure reflect changes in intrathoracic pressure and affect transpulmonary pressure, both of which have multiple effects on right and left ventricular performance. During passive breathing, increasing esophageal pressure is associated with lower venous return and higher right ventricular afterload and lower left ventricular afterload and oxygen consumption. In spontaneously breathing patients, negative pleural pressure swings increase venous return, while right heart afterload increases as in passive conditions; for the left ventricle, end-diastolic pressure is increased potentially favoring lung edema. Esophageal pressure monitoring represents a simple bedside method to estimate changes in pleural pressure and can advance our understanding of the cardiovascular performance of critically ill patients undergoing passive or assisted ventilation and guide physiologically personalized treatments.

## 1 Introduction

Breathing is accompanied by cyclic variations of intrathoracic pressure inducing changes in lung volumes. Since the heart is located inside the thoracic cavity, changes in intrathoracic pressure cyclically modify the pressure surrounding the heart chambers and thus their loading conditions (Cournand et al., 1947). Moreover, the pulmonary vasculature, interposed between the right and the left heart, is affected by variations of lung volume and pressure, which results in cyclic changes in right ventricular outflow impedance, cardiac output, and regional pulmonary blood flow (Vieillard-baron et al., 1999). These phenomena are referred to as “cardiopulmonary interactions”. Adverse cardiopulmonary interactions can compromise right and left heart function, oxygen delivery, and promote lung edema. Thus, they play a critical pathophysiological and clinical role in patients with the acute respiratory distress syndrome (ARDS).

## 2 Role of esophageal pressure to monitor cardiopulmonary interactions

Spontaneous and mechanical breaths induce cyclic changes in lung volume and the position of the chest wall during inspiration and expiration. Changes in volumes are accompanied by changes in the distending pressure of these structures. The distending (transmural) pressure of the chest wall is the pressure in the pleural space minus the atmospheric pressure, assumed to be 0 [i.e., the pleural pressure (Ppl)]. The distending pressure of the lungs is the transpulmonary pressure (P_L_), and corresponds to the difference between airway pressure and Ppl. Since the lungs and chest wall are in series, the total pressure needed to change the volume of the respiratory system [i.e., the airway pressure (Paw)] is the sum of the distending pressure of the lung plus the distending pressure of the chest wall. This total pressure is partitioned between the two components as a function of the relative elastances of the lung and chest wall (Cortes-Puentes et al., 2015) (O’Quin et al., 1985). Higher lung elastance (i.e., decreased lung compliance), for the same Paw, will result in higher P_L_, while a stiff chest wall will be reflected in increased Ppl. In clinical practice, esophageal pressure (Pes) is the most common method used for the measurement of Ppl. Pes allows calculation of P_L_ and the relative contributions of the lung and chest wall to the total stiffness of the respiratory system (Mauri et al., 2016a).

Measurement of Ppl is of great relevance for assessing heart-lung interactions. Indeed, Pes (i.e., Ppl) corresponds to the pressure surrounding the superior vena cava and the heart, which affects systemic venous return, transmural cardiac pressure, and cardiac output (Jardin et al., 1985); P_L_, instead, acts on the intrapulmonary vessels, potentially compressing them and affecting the afterload of the upstream right heart. Therefore, the cardiovascular consequences of breathing depend on the main alteration in the mechanical properties of the lungs vs. the chest wall, which can only be inferred through measurement of Pes. Of note, absolute values of Pes correspond to regional absolute values of Ppl in the dorsal region surrounding the heart, while Ppl will be lower in non-dependent regions.

## 3 Acute respiratory distress syndrome increases the risk for adverse cardiopulmonary interactions

Acute respiratory distress syndrome (ARDS) is defined by the presence of inflammatory lung edema leading to pulmonary infiltrates and impaired oxygenation (ARDS Definition Task Force; Ranieri et al., 2012). Changes in lungs and chest wall mechanics (Panwar et al., 2020), combined with functional and anatomical alterations of the pulmonary circulation (Bull et al., 2010) characterize the pathophysiology and clinical manifestation of ARDS.

Expiratory and inspiratory Paw are increased in ARDS, leading to increased P_L_ and Ppl. End expiratory positive Paw (PEEP), indeed, is essential for the diagnosis of ARDS, leading to increased Ppl at end expiration. Then, in spontaneously breathing patients, increased respiratory drive and effort (Spinelli et al., 2023) may result in large inspiratory decreases in Ppl (Tonelli et al., 2017). In passive patients, instead, mechanical ventilation increases the absolute values of Ppl during inspiration. In both cases, high inspiratory P_L_ could develop, especially in patients with increased lung elastance due to alveolar flooding, collapse, and de-recruitment (Mauri et al., 2016b).

Pulmonary vascular dysfunction often develops in the injured lung due to a combination of compression (Hakim et al., 1982), constriction (Marshall et al., 1994), and obstruction (Zapol and Jones, 1987) of pulmonary vessels. Because of its increased resistance and decreased distensibility, the pulmonary circulation has a reduced capacity to accommodate cyclic changes in blood flow that are due to changes in lung volume and P_L_, further increasing right heart afterload during inspiration. In addition, shifts in intravascular volume, which are characteristic of patients with ARDS, increases the risk for adverse heart-lung interactions in the presence of unphysiological Ppl (Berger et al., 2016). On the one hand, hypovolemia further amplifies the negative effects of increased Ppl on venous return and cardiac output (Vieillard-Baron et al., 2001). On the other, increased permeability of the alveolar-capillary barrier facilitates the accumulation of edema in response to large inspiratory decrease of Ppl, leading to excessive capillary transvascular pressure.

Given the complex and sometimes opposing effects of increased vs. decreased Pes during ventilation, we will now describe the pathophysiology of heart-lung interactions through the dynamics of Ppl and P_L_ during passive ventilation vs. spontaneous breathing. We will also describe how adverse interactions can precipitate heart failure and/or worsen lung injury.

## 4 Effects of transpulmonary and pleural pressure on the cardiovascular system during controlled mechanical ventilation

When muscular activity is abolished and the patient is passively ventilated, Pes increases during inspiration and decreases during expiration. The absolute value of Pes can be either positive or negative during the respiratory cycle, depending on airway pressure, the degree of lung edema, the patient’s body weight and relative chest wall and lung elastance.

### 4.1 Right heart

Pleural pressure is transmitted to the pericardium and therefore any increase in Pes due to passive ventilation changes the pericardial pressure and, consequently, the right heart transmural pressure. Venous return is driven by the gradient between mean circulatory filling pressure (Pmcf) and the right atrial pressure (Pra), which is usually only 4–8 mmHg (Guyton et al., 1957). During positive pressure ventilation, increased Pes can impact Pra and therefore venous return and cardiac output in two ways: during the respiratory cycle (increase in Pes related to tidal volume) or at the end of expiration (increase in baseline Pes by application of PEEP). Higher tidal volumes and PEEP will increase Pra, therefore reducing venous return, the end-diastolic volume and the stroke volume of the right ventricle.

Nevertheless, the negative effects of increased Pes are not linear and subject to compensatory mechanisms. Studies found that, in passively ventilated patients, higher PEEP and therefore higher end-expiratory Pes, can increase both Pra and Pmcf, without impacting the gradient for venous return (Van Den Berg et al., 2002). This could be related to the downward displacement of the diaphragm induced by PEEP, which could increase abdominal pressure and thus Pmcf, or to the presence of feedback by baroceptors that increase systemic venous tone (Fessler et al., 1991).

### 4.2 Pulmonary vasculature

The pulmonary circulation is affected by changes in lung volume and thus in P_L_. Lung inflation, which is associated with an increase in P_L_, has opposing effects on extra-alveolar and alveolar capillaries, expanding the first and compressing the latter. Thus, passive inspiration is associated with collapse of alveolar capillaries in the middle vascular segment (Marini et al., 2003).

In 1965, West described the interaction between pulmonary artery pressure (PAP), alveolar pressure (PA) and pulmonary venous pressure (Pv) identifying 3 zones with different driving pressure for blood flow and approximating its behavior to a Starling resistor (West and Dollery, 1965). A Starling resistor can be imagined as a compressible tube inside a box where flow is possible only when the entrance pressure (PAP) is higher than the box pressure (PA) and of the output pressure (Pv) (Permutt et al., 1962). In zone 1, PA is higher than PAP and therefore flow is zero; in zone 2, PAP is higher than both PA and Pv, flow is preserved but dependent on PA; in zone 3, PA is lower than both PAP and Pv, flow is present and independent of PA. In the presence of high lung elastance, elevated P_L_ can increase the amount of zone 1 and 2 (non-zone 3) conditions, thereby increasing right ventricular afterload during inspiration (Slobod et al., 2022). Although the normal RV is well adapted to handle cyclic small increases in afterload (Magder, 2021), many patients admitted to the ICU have RV contractile dysfunction or limitation of filling and cyclic tidal increases in RV afterload can have dramatic hemodynamic consequence (Magder et al., 2023).

Both high Ppl and P_L_ affect RV performance, but there is no consensus on which has the greater impact and computational modeling showed inconclusive results (Magder and Guerard, 2012). During controlled mechanical ventilation, the inspiratory increase in Ppl is responsible for the decreased preload effect, while the increase in P_L_ is associated with an increased afterload effect (Slobod et al., 2022) (Repessé et al., 2018) (Table 1). Both these effects generate reversed pulsus parodoxus (Massumi et al., 1973). In experimental models (Webb and Tierney, 1974) (Hotchkiss et al., 2001) (Katira et al., 2017) of severe pulmonary edema due to extremely high driving pressure (Webb and Tierney, 1974), right heart failure developed (Katira et al., 2017), likely because of combined preload and afterload effects, causing dramatic oscillations of pulmonary vascular resistance and blood flow with each breath.

**TABLE 1:** Summary of cardiopulmonary changes affecting the right ventricle during spontaneous versus passive ventilation in the setting of decreased and normal lung compliance.

Lung inflation	Tidal volume 6 mL/kg PBW			
	Decreased lung compliance		Normal lung compliance	
	Passive breath	Spontaneous breath	Passive breath	Spontaneous breath
Pleural pressure	↑	↓↓↓ (then ↑)	↑↑↑	↓ (then ↑↑↑)
Transpulmonary pressure	↑↑↑	↑↑↑	↑	↑
RV preload	↓	↑↑↑	↓↓↓	↑
RV afterload	↑↑↑	↑↑↑	↑	↑

### 4.3 Left ventricle

Positive pressure ventilation can impact LV performance through both direct and indirect effects. Indirectly, the effects of increased Pes and P_L_ on RV preload and afterload can reduce LV preload and stroke volume (Corp et al., 2021). RV dilation caused by an increase in afterload will also impact LV performance via ventricular interdependence (Weber et al., 1981).

Higher pleural pressure can also directly affect LV outflow. An increase in Pes simultaneously acts on the LV and aorta resulting in higher LV wall compression. Despite unchanged ventricular-aortic gradient, the absolute ventricular and aortic pressure will increase, as well as the gradient between thorax and peripheral vessel, leading to improved cardiac output. Moreover, the increased aortic wall pressure triggers autoregulation by peripheral baroreceptors causing a reduction of systemic vascular resistance and LV afterload (Rankin et al., 1982), further improving cardiac output. Finally higher Pes by positive pressure ventilation reduces the transmural LV gradient, decreasing oxygen consumption for the same absolute arterial pressure, thus protecting the stressed LV.

In summary, sustained increase of Pes during passive ventilation can improve or depress cardiac output (Table 1), depending on the patient’s characteristics. In patients with RV failure or preload dependence, cardiac output will decrease. On the contrary, in afterload dependent states (e.g., LV failure), higher pleural pressure can improve LV function and cardiac output (Alviar et al., 2018).

Another important aspect to be considered is the duration of exposure to a change in pleural pressure. Different components that play a role in influencing cardiac output do not have the same kinetics in response to sustained pleural pressure change. For example, it is well known that left ventricular outflow is affected by an inspiratory hold (Valsalva maneuver) only when pleural pressure is increased for a relevant length of time (e.g., >10 s) (Gavelli et al., 2019).

Finally, the effect of intrathoracic pressures on LV may not automatically influence the RV. Computational models (Magder and Guerard, 2012) showed that pulmonary vessels can act as a buffer, cyclically increasing and decreasing their blood content. This phenomenon may be dampened by large change in pleural pressure since increased transpulmonary pressure impacts transmural vascular pressure and reduce the capacitance for lung blood volume.

### 4.4 Distal organs

Increased pleural pressure due to PEEP during controlled mechanical ventilation may impact perfusion of distal organs, such as brain, liver and kidneys. For example, experimental studies showed that intrathoracic pressure can increase intracranial pressure (ICP), but the effect also depends on the relative elastances of the chest-wall and the lung. In conditions of high chest wall elastance, an increase of intrathoracic pressure may increase ICP (Chen et al., 2018). Increase in intrathoracic pressure has been shown to correlate also with a decrease in renal plasma flow, glomerular filtration rate (GFR) and urine output during mechanical ventilation. This is associated to a reduction of cardiac output (Annat et al., 1983) and an increase of the venous and parenchymal pressures. Further studies are needed to investigate the relative impact of pleural and/or transpulmonary pressure on distal organ function.

## 5 Effects of transpulmonary and pleural pressure on the cardiovascular system during spontaneous breathing

There is increasing clinical interest in maintaining spontaneous breathing in the care of patients with ARDS (Goligher et al., 2020). This strategy has cardiovascular benefits, but also potential harms, in particular in patients with RV dysfunction or limitation, in whom small changes in loading conditions can have important hemodynamic consequences (Magder et al., 2023). During assisted ventilation, a patient’s inspiratory effort induces a decrease in Pes, which adds to positive pressure provided from the ventilator, resulting in an increase P_L_ which inflates the lung (Bianchi et al., 2022). Monitoring Pes is the only way to quantify the inspiratory decrease in Ppl, which is otherwise “invisible” on the ventilator screen.

### 5.1 Preload and afterload effects

During an inspiratory effort, the Pes surrounding the RV falls, causing an increase in transmural pressure. This does not increase RV afterload because the downstream vascular bed into which the RV ejects (pulmonary arteries) is subject to the same change in pleural pressure. Moreover, the inspiratory fall in Pes increases the pressure difference between the Pmcf in the upstream systemic veins and the Pra (Magder, 2018). This favors venous return and will augment stroke volume if the right ventricle has not reached a state of limitation (Magder, 2018; Magder et al., 2023). Indeed, spontaneous breathing during mechanical ventilation has been associated with an increase in RV end diastolic volume and cardiac output, suggesting that preload is increased compared to passive ventilation (Rankin et al., 1982) (Masmoudi et al., 2017).

**FIGURE 1:**
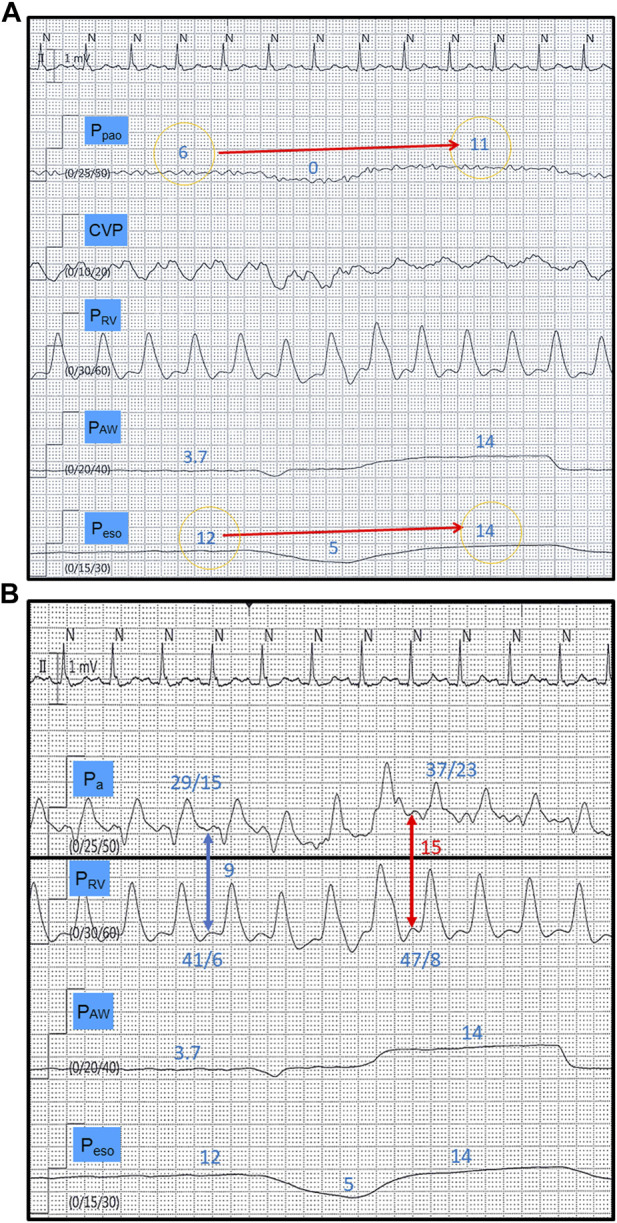
Tracings from a spontaneously breathing patient (CPAP mode) with increased lung elastance. The disproportionate end-inspiratory rise in P_pao_ during a breath hold maneuver (11 versus 6 mmHg) compared to P_eso_ (14 versus 12 mmHg) indicates development of West non-zone 3 conditions **(A)** (reference 23). This is translated into a 66% inspiratory increase in right ventricular afterload (red and blue arrows), measured as the right ventricular isovolumetric contraction pressure (diastolic P_a_–diastolic P_RV_) **(B)**. CPAP, continuous positive airway pressure, P_pao_, pulmonary artery occlusion pressure; CVP, central venous pressure; P_RV_, right ventricular pressure; P_AW_, airway pressure; P_eso_, esophageal pressure; P_a_, pulmonary artery pressure. All pressure values in mmHg.

Compared to passive ventilation, it must be emphasized that although Pes falls during the inspiratory phase of a spontaneous breath, P_L_ must increase to generate lung inflation. While the “preload effect” of changes in Ppl has differing consequences during passive ventilation (negative) and spontaneous breathing (beneficial), the cardiovascular effects of the inspiratory increase in P_L_ are similar, regardless of whether inspiratory efforts are present or not. Therefore, the increase in non-zone 3 conditions also occurs during spontaneous breathing, leading to increased afterload opposing RV ejection (Figure 1) (Hughes et al., 1968) (Brower et al., 1985) (Mekontso Dessap and Vieillard-Baron, 2022). This relationship between increasing inspiratory P_L_ and RV afterload has been demonstrated to be linear in patients with and without lung injury (Slobod et al., 2022) (Vieillard-baron et al., 1999).

The net effect of spontaneous breathing on cardiac output will again reflect the balance of these changes in RV preload and afterload. This balance will ultimately depend on the relative inspiratory change in Pes vs. P_L_ and on lung and chest wall mechanics, tidal volume, inspiratory effort, and volume status (Spinelli et al., 2020) (Repessé et al., 2018). Vigorous inspiratory effort in the setting of increased lung elastance results in increased P_L_ that will increase RV afterload due to increased non-zone 3 conditions. Moreover, unlike passive ventilation, large deflections in pleural pressure will augment venous return (Table 1). During subsequent RV ejections, increased stroke volume will be ejected into a pulmonary vasculature that is more extensively in non-zone 3, opposing RV ejection. In this way, vigorous efforts, coupled with low lung compliance, may increase shear forces and inflammation within the pulmonary vasculature.

### 5.2 Role of adverse cardiopulmonary interactions in the pathogenesis of LV failure

Unlike for the RV, decreases in Pes directly increase LV afterload due to the increase in transmural ventricular pressure. The inspiratory decrease in Pes represents an additional load that the LV must overcome to eject blood through the aortic valve. Increased inspiratory effort can thereby increase left ventricular end-diastolic pressure and facilitate cardiogenic pulmonary edema. Furthermore, a significant inspiratory reduction in Pes lowers the pressure surrounding most of the pulmonary vasculature, causing an increase in the transvascular pressure across the pulmonary capillary endothelium, worsening the risk of hydrostatic edema. Given that Pes deflections also increase venous return that can cyclically increase pulmonary perfusion and intravascular pressure, spontaneous breathing with increased inspiratory effort can favor fluid extravasation from pulmonary vessels, causing pulmonary edema (Spinelli et al., 2020) (Repessé et al., 2018).

In patients with ARDS, respiratory drive is frequently increased due to several mechanisms (Brochard et al., 2017), and vigorous inspiratory efforts can perpetuate a vicious cycle of impaired gas exchange, increased respiratory drive, and worsening lung edema. This process has been termed patient self-inflicted lung injury (Spinelli et al., 2020). Another clinical consequence of adverse heart-lung interactions in the context of high respiratory effort is “weaning induced pulmonary edema” for patients failing spontaneous breathing trials or extubation (Lhéritier et al., 2013; Liu et al., 2016).

## 6 Echocardiographic assessment of adverse cardiopulmonary interactions

Through the above listed mechanisms, mechanical ventilation may decrease RV preload or increase RV afterload. Each of these effects may lead to consistent or tidal decreases in stroke volume that can be identified by echocardiography. Changes in stroke volume may be evaluated by monitoring the velocity time integral, peak velocity and mean acceleration of flow across the pulmonic valve. A decrease in mean acceleration may more closely reflect an increase in RV afterload (Vieillard-baron et al., 1999; Slobod et al., 2022). Extreme consequences of mechanical ventilation on RV afterload may also be inferred by an increase in the RV to LV end-diastolic area ratio, with values >0.6 being suggestive of acute cor pulmonale (Rudski et al., 2010). Persistent exposure to increased RV afterload may, over time, result in RV dysfunction (decrease in end-systolic elastance) that may be evaluated with measurements of tricuspid annular motion and fractional area change of the RV (Rudski et al., 2010; Magder et al., 2023).

## 7 Bedside management of ventilated patients with hemodynamic instability

Management of hemodynamic failure in the setting of invasively ventilated patients with ARDS is a complex subject that needs close continuous monitoring. Clinical priorities include maintaining systemic and coronary perfusion while keeping filling pressures (namely, central venous pressure, CVP) within a physiologic range. Practically, this can be approached with inotropic support of the RV and reduction of RV afterload that may be achieved with optimization of mechanical ventilation to keep transpulmonary pressures as low as possible while maintaining adequate gas exchange. Positive pressure ventilation may induce a reduction in RV preload and fluid loading may be helpful to increase the mean systemic filling pressure and maintain venous return. However, it must be emphasized that to maintain a constant RV preload, that is, transmural CVP (CVP-Pes) in the setting of increasing pleural pressure, CVP relative to atmosphere must increase. Although this can be accomplished through fluid loading, clinicians must remain vigilant to avoid abrupt and sustained increases in CVP where possible, as increased CVP is associated with injury of other organs including the kidneys and liver (Pesenti et al., 2023). Increasing CVP with fluid loading without increasing stroke volume is a hallmark of RV limitation and is only associated with harm (Magder et al., 2023). Identification of RV limitation should prompt the clinician to halt fluid loading and consider alternative treatments to improve RV cardiac output (e.g., inhaled pulmonary vasodilators).

## 8 Conclusion

Adverse heart-lung interactions can complicate the clinical course of hypoxemic patients with ARDS at different clinical stages, facilitating the early progression of lung injury (both for non-intubated or passive patients) and impeding weaning from mechanical ventilation. Esophageal pressure monitoring allows precise assessment of the mechanical effects of pleural pressure and/or transpulmonary pressure on central hemodynamics and may be essential to predict, recognize and understand the pathophysiology of cardiopulmonary complications.
